# Risk Factors for Relapse in Antineutrophil Cytoplasmic Antibody–Associated Vasculitis Among Patients With Relapse After Induction of Remission With Rituximab

**DOI:** 10.1002/art.70025

**Published:** 2026-03-16

**Authors:** Ellen Romich, Joshua F. Baker, Thomas R. Riley, Ian Green, Rennie L. Rhee, Carol A. McAlear, Ulrich Specks, Rona M. Smith, David R. Jayne, Peter A. Merkel, for the RITAZAREM Investigators, Reem Al‐jayyousi, Yoshihiro Arimura, Jacqueline Andrews, Simon Bond, Marianna Nodale, Annette Bruchfeld, Brian Camilleri, Simon Carette, Chee Kay Cheung, Michael Clarkson, Janak de Zoysa, Vimal Derebail, Tim Doulton, Tomomi Endo, Eri Muso, Tatsuo Tsukamoto, Alastair Ferraro, Lindsy Forbess, Shouichi Fujimoto, Shunsuke Furuta, Ora Gewurz‐Singer, Yoshitomo Hamano, Lorraine Harper, Toshiko Ito‐Ihara, Rachel B. Jones, Nader Khalidi, Rainer Klocke, Curry L. Koening, Yoshinori Komagata, Hajime Kono, Shunya Uchida, Carol A. Langford, Peter Lanyon, Sarah Lawman, Raashid Luqmani, Larry W. Moreland, Kim Mynard, Patrick Nachman, Christian Pagnoux, Chen Au Peh, Charles Pusey, Dwarakanathan Ranganathan, Ken‐ei Sada, Richard Smith, Richard Watts, Robert Spiera, Antoine G. Sreih, Kazuo Suzuki, Vladamir Tesar, Augusto Vaglio, Giles Walters, Caroline Wroe

**Affiliations:** ^1^ Division of Rheumatology, Department of Medicine University of Pennsylvania Philadelphia; ^2^ Division of Epidemiology, Department of Biostatistics, Epidemiology, and Informatics University of Pennsylvania Philadelphia; ^3^ Division of Pulmonary and Critical Care Medicine Mayo Clinic Rochester Minnesota; ^4^ Department of Medicine University of Cambridge Cambridge United Kingdom; ^5^ Cambridge University Hospitals NHS Foundation Trust Cambridge United Kingdom

## Abstract

**Objective:**

The objective of the study was to determine risk factors for relapse of antineutrophil cytoplasmic antibody (ANCA)–associated vasculitis (AAV) after reinduction of remission with rituximab and discontinuation of maintenance therapy.

**Methods:**

This is a post hoc analysis of the RITAZAREM clinical trial. Patients aged 15 years or older with AAV and a positive test for anti–proteinase‐3 or anti‐myeloperoxidase‐ANCA who achieved remission after reinduction with rituximab and glucocorticoids were randomized at month 4 to receive continued rituximab or azathioprine for a maintenance period up to 24 months, followed by observation until relapse or up to 48 months. Generalized estimating equations logistic regression identified baseline and time‐varying risk factors for relapse by the next visit for the two study phases: maintenance (months 4–24) and off‐treatment (months 24–48).

**Results:**

Among 170 patients (median [interquartile range] age 59 [48–68] years, disease duration 5 [2–10] years), 99 relapses occurred (46 during maintenance). During maintenance, musculoskeletal involvement (odds ratio [OR]: 2.8, 95% confidence interval [CI]: 1.1–7.2; *P* = 0.03) and higher patient global assessment (OR: 1.1, 95% CI: 1.0–1.2; *P* = 0.04) were associated with relapse. During the off‐treatment phase, presence of CD19^+^ B cells (OR: 2.5, 95% CI: 1.2–5.1; *P* = 0.01) and reappearance of ANCA (OR: 3.2, 95% CI: 1.3–7.7; *P* = 0.01) were each associated with higher relapse risk. Multivariable analysis identified markers of inflammation (changes in platelets, white blood cells, and IgA) associated with relapse.

**Conclusion:**

Risk factors for relapse in AAV vary by treatment phase. Monitoring markers of inflammation and immune reconstitution may identify patients at risk for relapse, particularly after treatment withdrawal.

## INTRODUCTION

Antineutrophil cytoplasmic antibody (ANCA)–associated vasculitis (AAV), including granulomatosis with polyangiitis and microscopic polyangiitis, is a group of multisystem autoimmune diseases that often presents with organ‐ and life‐threatening manifestations and is treated with prolonged use of immunosuppressive medications. Approaches to induction of remission in new or relapsing disease and maintenance of remission have advanced considerably, resulting in improved survival.^1^, ^2^ Despite these advances, relapses are common during maintenance treatment and particularly once treatment is discontinued.^3^, ^4^ However, prolonged immunosuppression carries risks including secondary immunodeficiency, and not all patients experience relapse after completion of an induction and maintenance regimen.^5^ Optimal dosing and treatment duration is not known.

Prior studies demonstrated that factors associated with relapse in AAV include disease manifestations such as pulmonary, renal, and sinonasal disease; treatment phase (maintenance vs postmaintenance); return of a positive test for ANCAs or rising ANCA levels; return of detectable CD19^+^ B cells (peripheral blood); and patient‐reported outcomes.^6^, ^7^, ^8^, ^9^, ^10^, ^11^, ^12^, ^13^, ^14^, ^15^ Genetic polymorphisms may also influence response to treatments, such as response to rituximab with Fcγ receptor or B cell–activating factor variants.^16^, ^17^ However, the ability to predict relapses in clinical practice remains limited. Existing studies commonly evaluate relapse risk over a several‐year follow‐up period, whereas in clinical practice it may be more practical to identify patients at high short‐term risk of relapse if such predictions could guide treatment or monitoring strategies.

The RITAZAREM clinical trial enrolled patients with ANCA‐positive AAV with clinical relapse. Following reinduction and achievement of remission with rituximab, patients were randomized to rituximab every four months or daily azathioprine, for a total of two years of treatment.^18^ Patients were observed off treatment for an additional 12 to 24 months after completion of the maintenance regimen. Compared to azathioprine, rituximab reduced risk of relapse by 60%.^18^

The objective of this post hoc analysis of the RITAZAREM data set was to identify risk factors for short‐term relapse in patients with AAV after successful reinduction of remission with rituximab, focusing separately on risk factors for relapse during maintenance treatment and off‐treatment observation phases. This approach reflects the clinical scenario of desiring to know a patient's risk of relapse within a few months, which could influence treatment decisions in real time.

## METHODS

### Study design

This study is a post hoc analysis of the RITAZAREM clinical trial. The primary aim of this analysis was to determine predictors of disease relapse by the next study visit for two study phases as defined in the trial protocol: (1) treatment for maintenance of remission (months 4–24) and (2) off‐treatment observation (months 24–48).^19^ In RITAZAREM, all patients received rituximab, 375 mg/m^2^ per week for four weeks, at month 0 for treatment to re‐induce remission, and patients who achieved remission by month 4 were randomized to receive either rituximab or azathioprine with the goal of maintaining remission.^18^ During the maintenance phase, patients randomized to rituximab received doses (1,000 mg) at months 4, 8, 12, 16, and 20, and patients randomized to azathioprine (2 mg/kg per day) received this medication from month 4 through 24, with the drug tapered off from month 24 to month 27 (Supplementary Figure S1). For the current study, maintenance and off‐treatment phases were analyzed separately to test if there were distinct drivers of relapse for patients during maintenance therapy versus observation off‐treatment. Data will be shared upon reasonable request. See Appendix for RITAZAREM Trial Investigators.

### Study participants

Major inclusion and exclusion criteria for the RITAZAREM trial were as previously described.^19^ Briefly, patients were aged 15 years or older with a diagnosis of AAV, had a current or prior positive test for proteinase‐3 (PR3)‐ANCA or myeloperoxidase (MPO)‐ANCA, and experienced a disease relapse after previously achieving remission. Patients were included in this subanalysis if they completed treatment with rituximab for reinduction of remission in the RITAZAREM trial, achieved remission again by month 4, and moved on to maintenance treatment. All patients gave written, informed consent for trial participation, and de‐identified data were used for this study.

### Outcomes

The main outcome for this study was relapse, assessed as a binary outcome at each visit and defined as a return of disease activity by or at the subsequent study visit (3‐ to 6‐month interval). This was chosen to reflect the clinical scenario of desiring to know a patient's risk of relapse *before the next follow‐up visit*. Relapse was defined as in the RITAZAREM trial as the return or first appearance of at least one item on the Birmingham Vasculitis Activity Score for Wegener's granulomatosis (BVAS/WG).^20^

### Covariates

#### Variables collected at enrollment

Data collected at trial enrollment included age; sex; body mass index; disease duration; ANCA type (anti‐PR3 or anti‐MPO); cumulative disease manifestations by organ system (constitutional, mucous membranes, ear/nose/throat, lung, kidney, musculoskeletal, skin, neurologic, cardiac, gastrointestinal, other); relapse severity at trial entry (severe or nonsevere); prednisone regimen at induction (1 mg/kg or 0.5 mg/kg); prior treatment with rituximab, azathioprine, or cyclophosphamide; and cumulative prior rituximab exposure. Treatment assignment in the maintenance phase, with rituximab or azathioprine, was determined upon randomization at month 4 among patients who achieved remission after induction therapy.

Genotyping was performed for the *FCGR2A* R131H (G>A variant) in a subset of patients with adequate samples available. The A allele corresponds to the missense variant R131H that influences the function of the Fcγ receptor; the AA genotype has been associated with complete remission and earlier time to remission among rituximab‐treated patients with AAV.^16^ Therefore, R131H genotype (categorical) was evaluated with an interaction with treatment (see Supplementary Methods for further detail).

#### Variables collected throughout follow‐up period (time‐varying)

Data collected at enrollment and follow‐up visits included prednisone dose, body mass index (certain visits only), number of months since last rituximab dose, laboratory measures (white blood cell count, hemoglobin, platelets, creatinine, erythrocyte sedimentation rate, C‐reactive protein, alanine aminotransferase, IgG, IgM, and IgA), MPO and PR3 positivity (binary), MPO and PR3 level, CD19^+^ B cell counts or percentage, BVAS/WG, physician global assessment (0–10, higher is worse), patient global assessment (0–10, higher is worse), EuroQOL five dimensions questionnaire visual analogue Scale (0–100),^21^ Patient‐Reported Outcomes Measurement Information System (PROMIS) Fatigue (short‐form 4a v1.0 T‐score^22^), PROMIS Pain Interference (short‐form 4a v1.1 T‐score^23^), and PROMIS Physical Function (short‐form 4a v2.0 T‐score^24^). Laboratory values were converted/harmonized to common units. Change in laboratory values and patient‐reported outcomes was operationalized as the percentage change relative to the month 4 value (remission). As a sensitivity analysis based on a prior study demonstrating that changes in the patient global were predictive of relapse, absolute change in the patient global assessment from the prior visit was also evaluated.^8^

#### ANCA values

MPO‐ANCA or PR3‐ANCA positivity was determined at local sites during the trial. Additionally, MPO‐ANCA or PR3‐ANCA levels were determined semiquantitatively by a central laboratory (Mayo Clinic) using an automated addressable laser‐bead immunoassay (BioPlex 2200, Bio‐Rad) after completion of the trial.^25^ A positive value was ≥1.0 antibody index unit. ANCA positivity for this study was based on the central laboratory result when available and otherwise was based on the local trial result. This approach minimized missing data (ANCA positivity <1% missing). Because patients were identified to have one ANCA type (MPO or PR3) at trial enrollment and ANCA type was prespecified to be included in the models, ANCA positivity was defined as either MPO or PR3 positive. Change in ANCA positivity during follow‐up was categorized as negative to negative, negative to positive, positive to positive, or positive to negative compared to the prior visit.

#### CD19 ^+^ B cell values

CD19^+^ B cell results were determined at local sites during the trial and reported as cell counts and/or as a percentage of white blood cells. Cell counts were converted to common units (cells per 10^9^/L). Test results recorded as less than the lower limit of the assay were recoded as zero (ie, <0.01 cells per 10^9^/L was recoded as 0 and <1% recoded as 0). The presence of B cells (binary) was defined as either ≥0.01 cells × 10^9^/L or ≥1%, similar to prior studies.^6^, ^13^ When B cell presence was missing for a given visit but the preceding and subsequent visits had identical results (ie, both present or both absent), that result was carried forward to replace the missing value. With this approach, data regarding B cell presence were missing in <10% of visits.

### Statistical analysis

The statistical approach can be found in the Supplementary Methods. Enrollment characteristics and month 4 (remission) laboratory and outcome measure results were summarized. Characteristics at month 4 were compared among patients who did and did not experience relapse during trial follow‐up using *t*‐test, Wilcoxon rank‐sum test, or chi‐square test, as appropriate. To allow comparison of effect sizes across variables with different units of measurement, laboratory values and patient‐reported outcome measures were natural log‐transformed to fit a normal distribution and then standardized to month 4 remission values by subtracting the mean of the month 4 values and dividing by the SD of the month 4 values.

Logistic regression incorporating generalized estimating equations was used to identify risk factors for relapse by the next visit in each study phase following a prespecified modeling approach and including all available outcomes over time. For the maintenance phase analysis, data from months 4 to 20 were included, whereas for the off‐treatment phase analysis, data from months 24 to 42 were included. Regression coefficients from these models can be interpreted as the relative odds of relapse before or at the next study visit among those with the exposure compared to those without the exposure.

Enrollment characteristics (static variables) were assessed first for their association with relapse. Variables associated in univariate analyses (*P* < 0.10) were explored as potential predictors for a multivariable model. Treatment group and ANCA type were prespecified to be included given their clinical relevance. Variables with *P* < 0.05 in the multivariable model were retained to establish a *base model*.

Time‐varying predictors were then assessed by evaluating their association with relapse when added to the base model (see Supplementary Table S2 for variable list). Time‐varying measures with *P* < 0.10 were included as potential predictors. Finally, the full *multivariable model* retained only variables with *P* < 0.05. Prespecified interactions between treatment and month, between ANCA positivity and B cell presence/return, and between treatment and B cell presence/return were also explored. The inclusion of a treatment and month interaction allows time‐effects to vary by treatment, thereby accounting for differences in medication exposure over time between treatment groups.

Generalized estimating equations with logistic link function, robust SEs, and exchangeable correlation structure was used, accounting for repeated measures per patient. Alternative correlation structures were also explored. Variables in the full multivariable models were assessed for collinearity using variable inflation factors, and neither model had variable inflation factors >10. Analyses were conducted using Stata (StataCorp; 2023. *Stata Statistical Software: Release 18*. StataCorp LLC.).

## RESULTS

**Figure 1:**
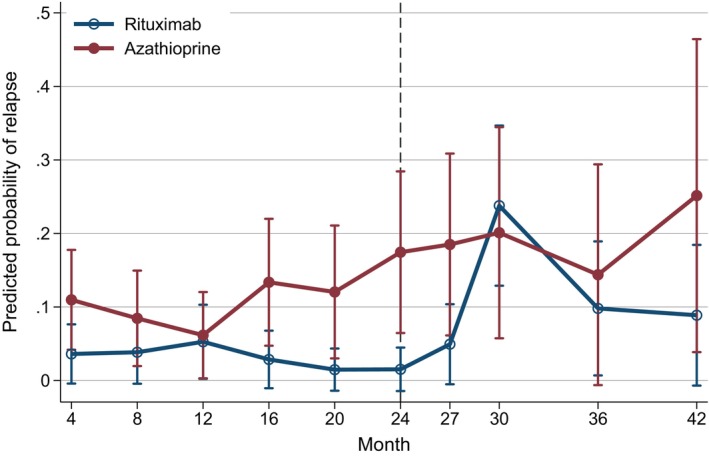
Predicted probability of relapse by month and treatment. Predicted probability of relapse by next visit from unadjusted generalized estimated equations logistic regression including treatment by month interaction. Probability of relapse is higher for the azathioprine group (red) at all time points except for month 30. The last scheduled rituximab maintenance treatment occurred at month 20 in the rituximab group (blue), while azathioprine was tapered off during months 24 to 27. The vertical line at month 24 divides the maintenance and off‐treatment phases for the analysis.

This study included 170 patients with a median (interquartile range) age of 59 (48–68) years and disease duration of 5 (2–10) years who were randomized to rituximab or azathioprine at month 4 after achieving remission (Table 1). During follow‐up, there were 99 relapses, with 46 during the maintenance phase (months 4–24) and 53 during the off‐treatment phase (months 24–48). Seventeen patients were censored due to death (n = 4), withdrawal (n = 8), or loss to follow‐up (n = 5). The probability of relapse by the next study visit by month and treatment increased over time and was higher for the azathioprine group at each time point except for between months 30 and 36 (Figure 1).

**Table 1:** Enrollment characteristics by relapse status*

	Total (N = 170)	Relapse (n = 99)	No relapse (n = 71)	*P* value
Age at enrollment, y	59 (48–68)	58 (46–67)	61 (53–72)	0.10
Female sex	86 (51)	47 (47)	39 (55)	0.34
Body mass index	28 (24–32)	29 (26–32)	26 (24–32)	0.02
Treatment group				<0.001
Rituximab	85 (50)	38 (38)	47 (66)	
Azathioprine	85 (50)	61 (62)	24 (34)	
Prednisone induction regimen				0.72
0.5 mg/kg/d	122 (72)	70 (71)	52 (73)	
1.0 mg/kg/d	48 (28)	29 (29)	19 (27)	
Relapse type at enrollment				0.38
Nonsevere	64 (38)	40 (40)	24 (34)	
Severe	106 (62)	59 (60)	47 (66)	
ANCA type at enrollment (ever)				0.06
anti‐MPO	47 (28)	22 (22)	25 (35)	
anti‐PR3	123 (72)	77 (78)	46 (65)	
Disease duration at enrollment, mo	5 (2–10)	4 (2–9)	6 (3–13)	0.04
Organ involvement (ever)				
Constitutional	91 (54)	52 (53)	39 (55)	0.76
Musculoskeletal	125 (74)	79 (80)	46 (65)	0.03
Skin	54 (32)	38 (38)	16 (23)	0.03
Mucous membranes/eyes	58 (34)	37 (37)	21 (30)	0.29
Ear/nose/throat	127 (75)	75 (76)	52 (73)	0.71
Cardiac	6 (4)	5 (5)	1 (1)	0.40
Gastrointestinal tract	3 (2)	2 (2)	1 (1)	1.00
Lungs	103 (61)	57 (58)	46 (65)	0.34
Kidneys	114 (67)	64 (65)	50 (70)	0.43
Nervous system	47 (28)	29 (29)	18 (25)	0.57
Other	40 (24)	28 (28)	12 (17)	0.08
Prior azathioprine treatment	126 (74)	75 (76)	51 (72)	0.56
Prior rituximab treatment	60 (35)	34 (34)	26 (37)	0.76
Cumulative rituximab dose before enrollment, g	3.9 (2.8–6.0)	4.0 (3.0–6.0)	3.0 (2.5–6.0)	0.43
Prior oral or IV cyclophosphamide	133 (78)	75 (76)	58 (82)	0.36
*FCGR2A* R131H genotype, n	128	50	78	
GG	39 (31)	16 (32)	23 (30)	0.93
AG	59 (46)	23 (46)	36 (46)	
AA	30 (23)	11 (22)	19 (24)	

* Values presented as n (%) or median (interquartile range) unless otherwise specified. ANCA, antineutrophil cytoplasmic antibody; *FCGR2A* R131H, Fcγ receptor IIa R131H variant; IV, intravenous; MPO, myeloperoxidase; PR3, proteinase‐3.

Patients who experienced a relapse at any time during follow‐up after randomization were more likely to receive azathioprine and have higher body mass index, shorter disease duration, and more frequent involvement of joints and skin (Table 2). They were also more likely to have lower serum creatinine, higher alanine aminotransferase, and higher IgG and IgA levels at month 4 (remission) (Supplementary Table S1).

**Table 2:** Univariable logistic generalized estimating equations models for odds of relapse by next visit for each phase using enrollment characteristics and study month*

Univariable models	Maintenance phase		Off‐treatment phase	
	OR (95% CI)	*P* value	OR (95% CI)	*P* value
Age at enrollment	0.99 (0.97–1.01)	0.30	1.00 (0.98–1.01)	0.71
Male (vs female)	0.90 (0.49–1.65)	0.74	1.58 (0.90–2.78)	0.11
Body mass index at enrollment^a^	1.49 (0.47–4.66)	0.50	3.05 (1.01–9.20)	0.05
Disease duration (years) at enrollment	0.91 (0.85–0.98)	0.01	0.98 (0.94–1.03)	0.50
Lung involvement	0.81 (0.44–1.48)	0.49	0.71 (0.40–1.26)	0.25
Ear, nose, throat involvement	0.94 (0.49–1.80)	0.85	1.17 (0.56–2.42)	0.68
Renal involvement	0.87 (0.46–1.63)	0.66	0.76 (0.42–1.37)	0.36
Musculoskeletal involvement	2.67 (1.12–6.39)	0.03	1.15 (0.61–2.19)	0.66
Skin involvement	1.16 (0.61–2.19)	0.65	1.77 (1.00–3.13)	0.05
Anti‐PR3 (vs anti‐MPO) at enrollment	0.97 (0.48–1.94)	0.92	1.68 (0.81–3.50)	0.16
Rituximab (vs azathioprine)	0.32 (0.17–0.62)	0.001	0.47 (0.27–0.83)	0.01
1.0 mg/kg/d (vs 0.5 mg/kg/d) induction prednisone regimen	1.27 (0.67–2.41)	0.46	1.03 (0.55–1.95)	0.93
Severe (vs nonsevere) relapse type at enrollment	0.62 (0.34–1.13)	0.13	0.90 (0.49–1.62)	0.72
BVAS/WG score at enrollment	1.00 (0.85–1.18)	0.98	1.10 (0.97–1.24)	0.14
Prior rituximab treatment	0.68 (0.35–1.32)	0.25	1.17 (0.66–2.08)	0.60
Cumulative prior rituximab dose (grams)	0.89 (0.79–1.00)	0.05	1.03 (0.96–1.11)	0.39
Prior azathioprine treatment	1.05 (0.53–2.06)	0.90	1.39 (0.70–2.74)	0.35
Prior oral or IV cyclophosphamide treatment	0.57 (0.29–1.10)	0.10	1.06 (0.50–2.24)	0.88
*FCGR2A* R131H genotype by treatment				
AG (vs GG), rituximab	1.71 (0.25–11.82)	0.59	2.85 (0.63–12.92)	0.18
AA (vs GG), rituximab^b^	–	–	4.11 (0.77–21.89)	0.10
Study month (vs mo 4)				
8	0.82 (0.33–2.00)	0.66	–	–
12	0.77 (0.30–1.94)	0.57	–	–
16	1.06 (0.44–2.55)	0.89	–	–
20	0.80 (0.31–2.11)	0.66	–	–
Study month (vs mo 24)				
27	–	–	1.29 (0.50–3.32)	0.60
30	–	–	3.33 (1.44–7.75)	0.005
36	–	–	1.47 (0.52–4.16)	0.46
42	–	–	1.89 (0.66–5.39)	0.23

* BVAS/WG, Birmingham Vasculitis Activity Score for Wegener's granulomatosis; CI, confidence interval; *FCGR2A* R131H, Fcγ receptor IIa; IV, intravenous; MPO, myeloperoxidase; OR, odds ratio; PR3, proteinase‐3.

^a^ Values log‐transformed.

^b^ Category omitted due to limited data.

### Maintenance phase

In unadjusted analyses, disease duration, musculoskeletal involvement (includes arthritis and/or arthralgia), cumulative prior rituximab dose, and prior cyclophosphamide treatment were associated with relapse (*P* < 0.1) (Table 2). Within the base model, disease duration at enrollment was associated with lower odds of relapse (odds ratio [OR]: 0.9, 95% confidence interval [CI]: 0.8–1.0; *P* = 0.005), whereas musculoskeletal involvement was associated with 2.8‐fold higher odds of relapse (OR: 2.8, 95% CI: 1.1–7.2; *P* = 0.029).

**Figure 2:**
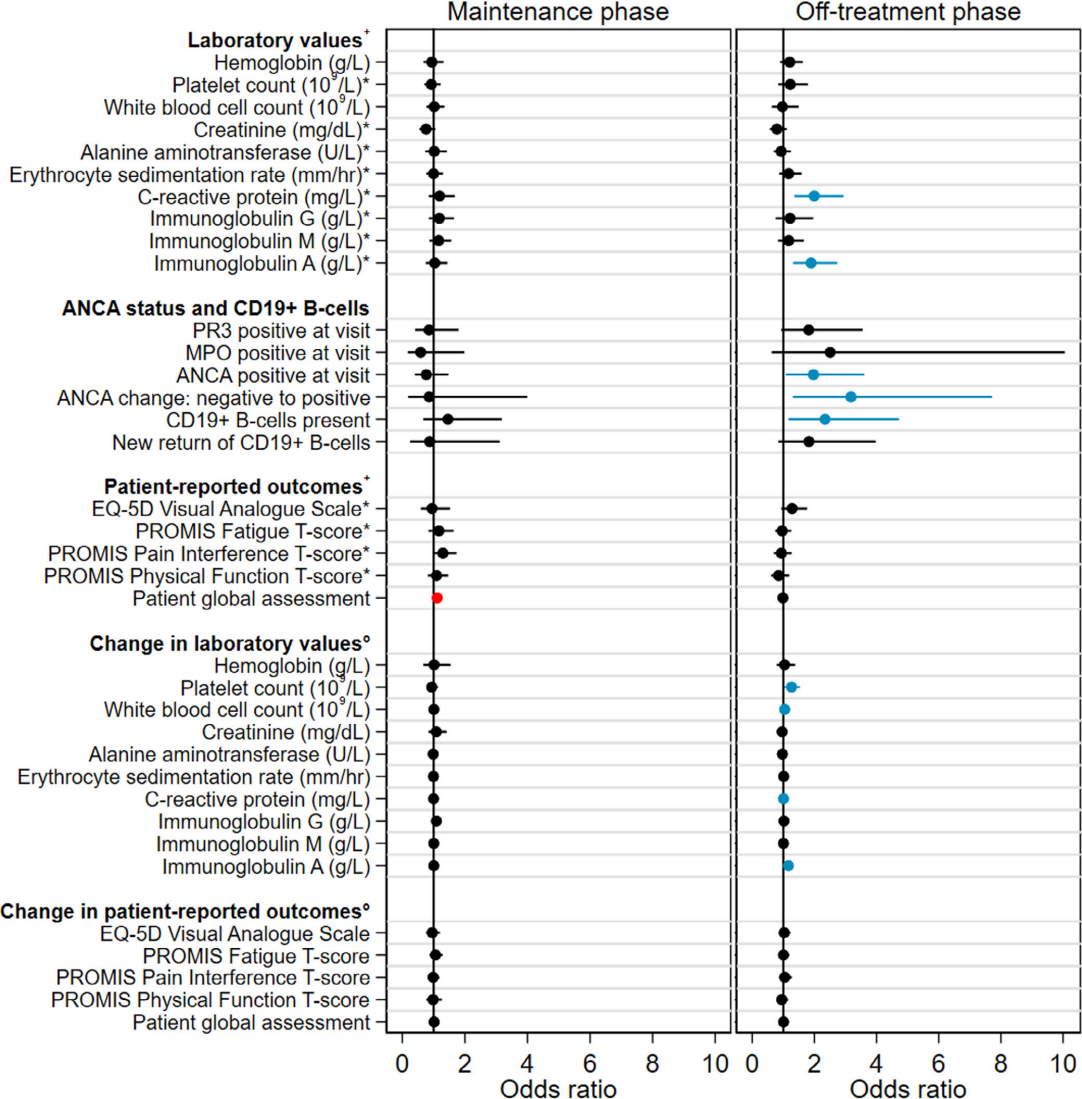
Odds ratios for relapse by next visit during the maintenance and off‐treatment phases for individual follow‐up variables. Odds ratios and 95% confidence intervals for relapse by next visit for laboratory values, ANCA status, and CD19^+^ B cells, patient‐reported outcomes, and change in these values compared to remission during the maintenance and off‐treatment phases. Each follow‐up variable was evaluated individually, and models were adjusted for the base model for each phase. Patient global assessment was associated with relapse by next visit during the maintenance phase (red). C‐reactive protein, IgA, ANCA positivity, change in ANCA from negative to positive, and CD19^+^ B cell presence are each associated with relapse by next visit during the off‐treatment phase (blue). ^+^Continuous values were standardized to month 4 (remission) values. *Indicates variable is log‐transformed. ^0^Odds ratios for change in laboratory values and patient‐reported outcomes are scaled to represent 10% change compared to remission. ANCA, antineutrophil cytoplasmic autoantibody; EQ‐5D, EuroQOL five dimensions questionnaire; MPO, myeloperoxidase; PR3, proteinase‐3; PROMIS, Patient‐Reported Outcomes Measurement Information System.

In univariate analyses for follow‐up variables, higher (worse) patient global assessment was associated with relapse (Figure 2; Supplementary Table S2). In the multivariable model, musculoskeletal involvement (OR: 2.8, 95% CI: 1.1–7.5; *P* = 0.038) and higher patient global assessment scores (OR: 1.1, 95% CI: 1.0–1.2; *P* = 0.043) were associated with higher odds for relapse, whereas treatment with rituximab (OR: 0.3, 95% CI: 0.2–0.7; *P* = 0.001) and longer disease duration were associated with lower odds for relapse (OR: 0.9, 95% CI: 0.9–1.0; *P* = 0.018) (Table 3). Laboratory values, ANCA status, CD19^+^ B cells, and *FCGR2A* R131H genotype were not associated with relapse during this phase. Percentage change in patient global assessment from month 4 (remission) and absolute change in patient global from the prior visit were not associated with relapse by next visit during the maintenance phase.

**Table 3:** Full multivariable logistic generalized estimating equations model for odds of relapse by next visit during observation and maintenance phases*

	Maintenance phase		Off‐treatment phase	
	OR (95% CI)	*P* value	OR (95% CI)	*P* value
Enrollment variables				
Treatment assignment: Rituximab (vs azathioprine)	0.3 (0.2–0.7)	<0.001	0.1 (0.0–0.6)	0.02
ANCA type at enrollment: anti‐PR3 (vs anti‐MPO)	0.9 (0.4–2.0)	0.80	1.7 (0.8–3.7)	0.20
Disease duration (years) at enrollment	0.9 (0.9–1.0)	0.02	–	–
Musculoskeletal involvement	2.8 (1.1–7.5)	0.04	–	–
Month (vs mo 24)				
27	–	–	1.0 (0.3–3.3)	0.95
30	–	–	0.8 (0.2–3.4)	0.81
36	–	–	0.4 (0.1–2.3)	0.28
42	–	–	0.9 (0.2–4.6)	0.91
Treatment by month interaction				
Rituximab, mo 27	–	–	1.3 (0.1–29.8)	0.88
Rituximab, mo 30	–	–	26.8 (1.7–428.3)	0.02
Rituximab, mo 36	–	–	21.1 (0.9–496.2)	0.06
Rituximab, mo 42	–	–	3.8 (0.2–73.1)	0.37
Follow‐up variables				
Patient global assessment (0–10)	1.1 (1.0–1.2)	0.04	–	–
IgA level (g/L)^a^	–	–	1.6 (1.1–2.4)	0.02
Percent change in IgA level (g/L) from month 4^b^	–	–	1.1 (1.0–1.2)	0.004
Percent change in platelet count (10^9^/L) from month 4^b^	–	–	1.2 (1.0–1.5)	0.02
Percent change in white blood cells (10^9^/L) from month 4^b^	–	–	1.04 (1.0–1.1)	0.04

* ANCA, antineutrophil cytoplasmic antibody; CI, confidence interval; MPO, myeloperoxidase; OR, odds ratio; PR3, proteinase‐3.

^a^ Values log‐transformed and standardized to month 4.

^b^ Odds ratio scaled to represent 10% change in value.

### Off‐treatment phase

In unadjusted analyses, higher body mass index and skin involvement at enrollment were associated with higher odds of relapse by the next visit. Additionally, relapses were more common at month 30 (OR: 3.3, 95% CI: 1.4–7.8; *P* = 0.005) (Table 2). There was a higher proportion of patients with relapse at month 30 in the rituximab group; therefore, an interaction between treatment and study month was included. The interaction suggested that the risk of flare was higher at month 30 among patients in the rituximab arm, but not in the azathioprine arm (*P* = 0.019 for interaction) (Table 3). Thus, the base model for the off‐treatment phase includes treatment assignment, ANCA type (both prespecified), study month, and the interaction between treatment and month.

Time‐varying follow‐up variables evaluated included laboratory values, patient‐reported outcome measures, and the percentage change in these variables compared to month 4 (remission). Results are shown in Figure 2 and Supplementary Table S2. Individually, higher C‐reactive protein, IgA, and percentage change in platelet count, white blood cell count, IgA, and C‐reactive protein were associated with increased odds of relapse by next visit (*P* < 0.05). Additionally, ANCA positivity (OR: 2.0, 95% CI: 1.1–3.6; *P* = 0.028), change in ANCA from negative to positive compared to the prior visit (OR: 3.2, 95% CI: 1.3–7.7; *P* = 0.011), and CD19^+^ B cell presence (OR: 2.5, 95% CI: 1.2–5.1; *P* = 0.017) were each associated with two‐ to three‐fold increased risk of relapse by the next visit during the off‐treatment phase, independent of treatment arm. The final full multivariable model for the off‐treatment phase included variables in the base model (treatment, ANCA type, study month, and treatment and month interaction), along with IgA level and changes (percentage) in IgA, platelet count, and white blood cells from month 4 (Table 3). ANCA status and CD19^+^ B cell presence were not significant in the full multivariable model. The patient global assessment and percentage change in the global assessment compared to month 4 were not associated with relapse during the off‐treatment phase. In a sensitivity analysis, the change in the patient global assessment from the prior visit during the off‐treatment phase was associated with relapse in unadjusted analyses (OR: 1.14, 95% CI: 1.02–1.28; *P* = 0.019) but was not significant in the multivariable model.

There was no interaction between treatment and the presence of CD19^+^ B cells in the off‐treatment phase (*P* = 0.109). Similarly, there was no significant interaction between treatment and ANCA positivity (in place of ANCA change) (*P* = 0.490). Replacing ANCA positivity (vs negativity) with ANCA level during the modeling process did not substantially change results.

As a sensitivity analysis, stratification by treatment group for the off‐treatment phase was explored. In the rituximab group, adjusting for ANCA type and month, CD19^+^ B cell presence was associated with relapse by the next visit (OR: 5.3, 95% CI: 1.5–19.4; *P* = 0.01), whereas ANCA positivity was not (OR: 1.6, 95% CI: 0.7–4.1; *P* = 0.292). In contrast, in the azathioprine group, ANCA positivity was associated with relapse (OR: 2.4, 95% CI: 1.0–5.9; *P* = 0.047), whereas CD19+ B cell presence was not (OR: 1.5, 95% CI: 0.6–3.5; *P* = 0.389). Due to small sample sizes, treatment‐stratified models with additional variables and time interactions were not feasible.

## DISCUSSION

This study identified risk factors for relapse after reinduction treatment with rituximab in patients with AAV at high risk of further relapse and identified distinct risk factors that differ according to each treatment phase. Shorter disease duration, musculoskeletal involvement, and higher (worse) patient global assessment were associated with relapse by the next visit during maintenance treatment. Markers of inflammation and immune reconstitution, meaning the presence or reappearance of serologic measures of systemic inflammation (eg, acute phase reactants), autoantibodies, and B cell function, were associated with relapse during the off‐treatment phase. In the full multivariable model, these include higher IgA level and an increase in IgA level, platelet count, and white blood cell count compared to remission values. Overall, these findings suggest that monitoring of these laboratory values to identify patients at higher risk of short‐term relapse may be most informative after treatment withdrawal.

Notably, there was a much higher risk of relapse by the next visit at study month 30 among patients who had been receiving maintenance rituximab (last maintenance dose administered at month 20) compared to azathioprine (tapered off during months 24–27). Patients receiving rituximab had 27‐times higher odds of relapse between month 30 and 36, although the wide CI indicates uncertainty about the true magnitude of increased odds. The potential for relapse may have differed owing to more patients with early relapse in the azathioprine group and continued azathioprine exposure during months 24 to 27. Nevertheless, this finding supports that withdrawal of treatment is significantly associated with relapse and highlights a high‐risk timeframe for relapse in patients treated with rituximab for maintenance of remission (approximately 10–16 months after the last infusion). This timeframe coincides with B cell repopulation after treatment with rituximab.^26^

The full models in the off‐treatment phase also suggest that immune reconstitution is a strong predictor of flare. Several variables were associated with relapse in univariate analyses, including higher C‐reactive protein, increases in C‐reactive protein compared to remission, presence of detectable CD19^+^ B cells, ANCA positivity, change in ANCA from negative to positive compared to the prior visit, and change in the patient global assessment from the prior visit. Although not all of these factors were retained in the final multivariable model, likely in part due to the interrelatedness of these measures as markers of inflammation and immune activity and a lack of granular assessments of B cell populations and ANCA levels over time, these findings provide further support that markers of immune reconstitution and inflammation are associated with short‐term relapse risk. Thus, multiple approaches to identifying patients with immune reconstitution may be informative.

Prediction of relapse in AAV is an area of considerable interest, with prior attention given to the utility of ANCA levels and CD19^+^ B cell counts.^9^, ^12^, ^27^, ^28^ This study identified associations between each of these measures and relapse in the off‐treatment phase but not the maintenance phase. CD19^+^ B cell repopulation coincides temporally with loss of drug effect. However, these measures did not remain in the final multivariable models, meaning that an independent predictive value was not found for the presence of CD19^+^ B cells or ANCA positivity above what could be predicted from the timing of dosing. The findings that IgA, C‐reactive protein, and white blood cell counts are associated with relapse are consistent with risk factors identified from the MAINRITSAN clinical trials.^29^ However, other commonly recognized risk factors for relapse, such as lung involvement, sinonasal disease, and anti‐PR3 antibody type, were not redemonstrated.^14^ Differences between studies may reflect distinct patient populations. The patient population from the current study was selected to include ANCA‐positive individuals with known relapsing disease. By enriching for relapsing disease and ANCA positivity, baseline disease manifestations differ from other trial populations that included patients with relapsing or nonrelapsing disease.^29^ The current study also confirms an association between patient global assessment of disease activity and subsequent relapse.^8^

Strengths of this study include standardized prospective data collection at international expert centers within a clinical trial setting, randomized treatment assignment, minimal loss to follow‐up, and centralized ANCA testing. A sequential modeling approach identified predictors with incremental benefit in the model while avoiding overfitting. Limitations to consider include generalizability from a clinical trial setting with selective enrollment of patients. Additionally, defining on‐ and off‐treatment phases poses challenges. For this analysis, treatment phases were based on a two‐year maintenance treatment period defined by the trial protocol. However, the end of rituximab's biologic effect is challenging to define, and patients receiving azathioprine tapered the medication off over months 24 to 27. Inclusion of a treatment and month interaction term in the off‐treatment phase models allows for time‐dependent differences in relapse likelihood between treatment groups, which may help to mitigate confounding arising from differences in medication exposure. Missing data were encountered for some laboratory values, particularly for CD19^+^ B cell measures, which were available as counts, percentages, or both. This limited the ability to include a single quantitative measure of B cells. Therefore, presence of B cells was defined based on thresholds for cell counts or percentages to accommodate the available data and reflect the ways B cell values are commonly encountered in practice. Similarly, although ANCA status was available from the trial data with minimal missingness, ANCA levels were determined separately at a central laboratory and only available for a subset of patients. However, in a sensitivity analysis, replacing ANCA status with quantitative ANCA binding level in the model building process yielded similar results.

These findings demonstrate that risk factors for short‐term relapse in AAV differ by phase of treatment and suggest that laboratory monitoring at regular intervals for markers of immune activity (acute phase reactants, immunoglobulins, ANCA status, CD19^+^ B cells) may be most informative after withdrawal of maintenance treatment. These results indicate that none of these tests in isolation is highly predictive of relapse. These findings highlight the efficacy of rituximab for maintenance treatment and the increased risk of relapse after treatment withdrawal, which can inform clinical practice. Additional longitudinal studies are needed to develop predictive tools to identify patients with increased risk of relapse over a short period and determine clinically significant thresholds of change in laboratory and immunologic parameters that influence risk of relapse. This may inform treatment and monitoring decisions, particularly after withdrawal of maintenance treatment. A prior study evaluated extended dosing of rituximab after completion of two years of fixed‐schedule maintenance rituximab using return of B cells versus rise in ANCA levels to determine when to readminister rituximab and found that administration based on B cell repopulation resulted in fewer clinical relapses but also more frequent infusions of rituximab and COVID‐19 infections.^13^ Consideration of other markers of immune activity may help to tailor management of individual patients after maintenance treatment. Studies to predict risk of treatment‐related infections will also be important to balance the risks and benefits of different approaches to maintenance treatment and observation after treatment withdrawal.

In conclusion, this study identified that among patients with AAV, markers of inflammation and immune reconstitution are associated with disease relapse within three to six months during the period of observation off treatment after completion of a regimen to maintain remission. These data support monitoring of these laboratory tests at regular intervals to help identify patients with AAV at risk for relapse after withdrawal of treatment.

## AUTHOR CONTRIBUTIONS

All authors contributed to at least one of the following manuscript preparation roles: conceptualization AND/OR methodology, software, investigation, formal analysis, data curation, visualization, and validation AND drafting or reviewing/editing the final draft. As corresponding author, Dr Romich confirms that all authors have provided the final approval of the version to be published, and takes responsibility for the affirmations regarding article submission (eg, not under consideration by another journal), the integrity of the data presented, and the statements regarding compliance with institutional review board/Declaration of Helsinki requirements.

## ROLE OF THE STUDY SPONSOR

Roche/Genentech had no role in the study design or in the collection, analysis, or interpretation of the data or the decision to submit the manuscript for publication. Publication of this article was not contingent upon approval by Roche/Genentech.

## Supporting information


**Disclosure Form**:

**Data S1.** Supporting Information.

## APPENDIX A RITAZAREM Trial Investigators

Collaborators of the RITAZAREM Trial Investigators are as follows: Reem Al‐jayyousi (Nephrology, University Hospitals of Leicester NHS Trust, Leicester, United Kingdom); Yoshihiro Arimura (Kyorin University School of Medicine, Tokyo, Japan); Jacqueline Andrews (NIHR Leeds Musculoskeletal Biomedical Research Unit, Leeds Teaching Hospitals Trust, Leeds, United Kingdom); Simon Bond and Marianna Nodale (Cambridge Clinical Trials Unit, Cambridge University Hospitals NHS Foundation Trust, Cambridge, United Kingdom); Annette Bruchfeld (Nephrology, Karolinska University Hospital and Karolinska Institute, Stockholm, Sweden); Brian Camilleri (Nephrology, Ipswich Hospital NHS Trust, Ipswich, United Kingdom); Simon Carette (Rheumatology, University of Toronto, Toronto, Ontario, Canada); Chee Kay Cheung (Nephrology, University of Leicester, Leicester, United Kingdom); Michael Clarkson (Cork University Hospital, Cork, Ireland); Janak de Zoysa (North Shore Hospital, Auckland, New Zealand); Vimal Derebail (Medicine, University of North Carolina at Chapel Hill, Chapel Hill, North Carolina); Tim Doulton (Nephrology, East Kent Hospitals University NHS Foundation Trust, Canterbury, United Kingdom); Tomomi Endo, Eri Muso, and Tatsuo Tsukamoto (Kitano Hospital, Osaka, Japan); Alastair Ferraro (Nephrology, Nottingham University Hospitals NHS Trust, Nottingham, United Kingdom); Lindsy Forbess (Rheumatology, Cedars‐Sinai Medical Center, Los Angeles, California); Shouichi Fujimoto (Hemovascular Medicine and Artificial Organs, Faculty of Medicine, University of Miyazaki, Miyazaki, Japan); Shunsuke Furuta (Allergy and Clinical Immunology, Chiba University, Chiba, Japan); Ora Gewurz‐Singer (Rheumatology, University of Michigan, Ann Arbor, Michigan); Yoshitomo Hamano (Tokyo Metropolitan Geriatric Hospital, Tokyo, Japan); Lorraine Harper (Nephrology, University of Birmingham, Birmingham, United Kingdom); Toshiko Ito‐Ihara (The Clinical and Translational Research Center, Kyoto Prefectural University of Medicine, Kyoto, Japan); Rachel B. Jones (Medicine, University of Cambridge, Cambridge, United Kingdom); Nader Khalidi (Medicine, McMaster University, Hamilton, Ontario, Canada); Rainer Klocke (Rheumatology, Dudley Group of Hospitals NHS Trust, Dudley, United Kingdom); Curry L. Koening (Rheumatology, The University of Utah, Salt Lake City, Utah); Yoshinori Komagata (First Department of Internal Medicine, Kyorin University School of Medicine, Tokyo, Japan); Hajime Kono and Shunya Uchida (Teikyo University Hospital, Tokyo, Japan); Carol A. Langford (Rheumatic and Immunologic Diseases, Cleveland Clinic Foundation, Cleveland, Ohio); Peter Lanyon (Rheumatology, Nottingham University Hospital, Nottingham, United Kingdom); Sarah Lawman (Brighton Royal Sussex County Hospital, Brighton, United Kingdom); Raashid Luqmani (Nuffield Department of Orthopaedics, Rheumatology and Musculoskeletal Science, University of Oxford, Oxford, United Kingdom); Larry W. Moreland (Medicine/Rheumatology, University of Pittsburg, Pittsburg, Pennsylvania); Kim Mynard (Vasculitis and lupus clinic, Cambridge University Hospitals NHS Foundation Trust, Cambridge, United Kingdom); Patrick Nachman (UNC Kidney Center, Division of Nephrology and Hypertension, University of North Carolina at Chapel Hill, Chapel Hill, North Carolina); Christian Pagnoux (Mount Sinai Hospital, University of Toronto, Toronto, Ontario, Canada); Chen Au Peh (Nephrology, Royal Adelaide Hospital, Adelaide, South Australia, Australia); Charles Pusey (Medicine, Imperial College London, London, United Kingdom); Dwarakanathan Ranganathan (Medicine, Royal Brisbane and Women's Hospital, Herston, Queensland, Australia); Ken‐ei Sada (Okayama University, Okayama, Japan); Richard Smith and Richard Watts (Ipswich Hospital NHS Trust, Ipswich, United Kingdom); Robert Spiera (Rheumatology, Hospital for Special Surgery, New York, New York); Antoine G. Sreih (Rheumatology, University of Pennsylvania Perelman School of Medicine, Philadelphia, Pennsylvania); Kazuo Suzuki (Teikyo University, Tokyo Japan); Vladamir Tesar (Medicine, Charles University, Praha, Czech Republic); Augusto Vaglio (University of Parma, Parma, Italy); Giles Walters (Nephrology, Australian National University Medical School, Canberra, Australian Capital Territory, Australia); and Caroline Wroe (Nephrology, South Tees Hospitals NHS Foundation Trust, Middlesbrough, United Kingdom).
