# Supplementary material for: Risk Factors for Relapse in Antineutrophil Cytoplasmic Antibody–Associated Vasculitis Among Patients With Relapse After Induction of Remission With Rituximab
Source: Arthritis Rheumatol. 2026 Mar 16;78(5):1134–44. doi: 10.1002/art.70025 (PMC13129626; doi:10.1002/art.70025)
Supplement: Supplementary file 2 — Data S1. Supporting Information. [file ART-78-1134-s002.docx]

**Supplementary Material - Table of Contents**

**Supplementary Methods:** Additional details on methods used in the study Page 2

**Supplementary Figure S1:** RITAZAREM study design Page 4

**Supplementary Table S1:** Laboratory values and outcome measures at month 4
(remission) according to relapse status Page 5

**Supplementary Table S2:** Logistic generalized estimating equations models for odds of
relapse by next visit for follow-up variables adjusted for the base model for each phase Page 6

**Supplementary References** Page 8

**Supplementary Methods**

*Genotyping and analysis of genetic data*

Genotyping was performed previously at the University of Toronto using the Affymetrix Biobank 1 Assay (Santa Cruz, CA). *FCGR2A* rs1801274 (R131H, A>G) is directly measured on this assay and passed quality control measures including Hardy-Weinberg equilibrium. Genotypes were called and processed using Affymetrix Genotyping Console version 4.2 and SNPolisher software. Quality control filtering on the full array was performed using Golden Helix SVS software. Samples from 128 patients were available for analysis after quality control measures were completed.

An important confounder in genetic epidemiology is the population structure; the differences in allele frequency related to the genetic ancestry of participants may lead to the identification of associations based on genetic ancestry rather than the effect of the variants of interest. As genetic ancestry cannot be directly measured, principal component analysis on the full quality-controlled genotypes was performed using Plink v1.9 to identify major clusters of genetic similarity in order to approximate genetic ancestry. The first nine principal components (PCs) explained > 90% of the variance and were selected for use as covariates in statistical analysis.

Genetic effects were modeled in the following ways: additive (dose-response, GG (reference)🡪 AG🡪 AA modeled as a continuous variable), genotype (categorical variable with reference GG), and autosomal recessive (AA compared to AG/GG as a binary variable) based on differing effects observed in the literature for the pharmacogenetic effects of this variant^1^. Five models were used for the analysis of the *FCGR2A* single nucleotide polymorphism: 1) unadjusted, 2) adjusted for principal components of population structure, 3) adjusted for age, sex, and principal components of population structure, 4) adjusted for treatment group, 5) interaction testing with treatment group. Effect sizes for the genetic variant in the unadjusted models and in models adjusted for principal components of population structure with or without age and sex were similar, suggesting that genetic ancestry does not meaningfully impact this variant in this population. Therefore, the principal components were not included in subsequent models.

*ANCA values*

MPO- or PR3-ANCA levels were determined semi-quantitatively by a central laboratory (Mayo Clinic) using an automated addressable laser-bead immunoassay (BioPlex 2200, Biorad) after completion of the trial^2^. MPO or PR3 binding levels ranged from < 0.2 to > 8.0 antibody index (AI) units. For analysis, values of <0.2 AI were re-coded as 0.1 AI and values of >8.0 AI were re-coded as 9.0 AI to distinguish values at vs. above or below the detection limits of the assay.

*Statistical analysis*

To allow comparison of effect sizes across variables with different units of measurement, laboratory values and patient-reported outcome measures were log-transformed to fit a normal distribution and then standardized to month 4 remission values by subtracting the mean of the month 4 values and dividing by the standard deviation of the month 4 values. Additionally, to aid interpretation of effect sizes, results for the percent change in laboratory and patient-reported outcome variables compared to month 4 were re-scaled by multiplying the regression coefficient by 10 such that the odds ratio represents the odds of relapse for a 10% change in the variable compared to the month 4 value. In a sensitivity analysis, absolute change in patient global assessment from the prior visit was included.

Logistic regression incorporating generalized estimating equations was used to identify risk factors for relapse by the next visit in each study phase following a pre-specified modeling approach. For the maintenance phase analysis, data from months 4-20 were included, while for the off-treatment phase analysis, data from months 24-42 were included. Longitudinal modeling approaches provided the ability to incorporate all outcomes in a single model. Regression coefficients from these models can be interpreted as the relative odds of relapse before or at the next study visit among those with the exposure.

Enrollment characteristics (static variables) were assessed first for their association with relapse (see Table 2 for variable list). Variables associated in univariate analyses (p < 0.10) were explored as potential predictors for a multivariable model. Treatment group and ANCA type were pre-specified to be included regardless of their univariate association given their clinical relevance. Study month was assessed in this step to account for time. A treatment by month interaction was identified for the off-treatment phase and was including in the base model. Variables were added one-by-one in order of smallest to largest p-value in univariate analyses, and those with p < 0.05 in the multivariable model were retained to establish the base model for each phase.

Time-varying predictors were assessed by evaluating their association with relapse when added to the base model (see Supplementary Table S2 for variable list). Time-varying measures with p < 0.10 were included as potential predictors in order of smallest to largest p-value. The full multivariable model retained only variables with p < 0.05 to establish the full model. Pre-specified interactions between treatment and month, ANCA positivity and B-cell presence/return, and treatment and B-cell presence/return were also explored in the full multivariable model.

In a sensitivity analysis, stratification by treatment group was explored for the off-treatment phase. A limited set of variables including ANCA positivity and CD19+ B-cell presence were evaluated based on clinical interest, with adjustment for ANCA type and study month. Due to small sample sizes, inclusion of additional variables or time-based interaction terms was not feasible. The primary analysis includes a treatment by month interaction term to account for time-based differences in relapse likelihood between treatment groups.

Generalized estimating equations with logistic link function, robust standard errors, and exchangeable correlation structure was used, accounting for repeated measures by patient. An unstructured correlation structure was also explored for the full models and results were unchanged. Variables in the full multivariable models were assessed for collinearity using variable inflation factor and neither model had variable inflation factors >10. Analyses were conducted using STATA (StataCorp. 2023. *Stata Statistical Software: Release 18*. College Station, TX: StataCorp LLC.).


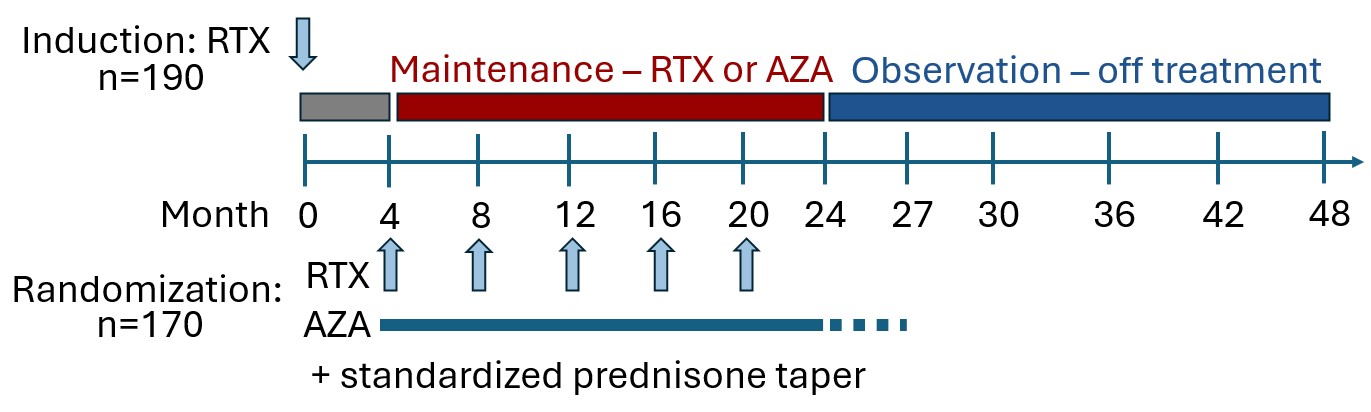


**Supplementary Figure 1. RITAZAREM study design**

Patients enrolled in RITZAREM (n=190) received rituximab for induction of remission at month 0. Patients in remission at month 4 (n=170) were randomized to continued rituximab (months 4, 8, 12, 16, 20) or daily azathioprine (months 4-24 with taper off by month 27) and received a standardized prednisone taper. Patients were observed off-treatment up to month 48. For this post-hoc analysis, maintenance phase is defined as months 4-24, while the off-treatment phase is defined as months 24-48, consistent with trial definitions^3^.

| **Supplementary Table S1. Laboratory values and outcome measures at month 4 (remission) according to relapse status** | | | | | |
| --- | --- | --- | --- | --- | --- |
|  | **Relapse (n = 99)** | | **No relapse (n = 71)** | | **p-value** |
| **Laboratory values** | **n** |  | **n** |  |  |
| Hemoglobin (g/L) | 98 | 132 (121-144) | 67 | 132 (120-139) | 0.37 |
| Platelet count (10^9^/L) | 98 | 261 (222-313) | 67 | 263 (211-307) | 0.63 |
| White blood cell count (10^9^/L) | 98 | 9.8 (7.6-11.3) | 67 | 9.4 (7.6-11.5) | 0.79 |
| Creatinine (mg/dl) | 98 | 1.1 (0.9-1.7) | 69 | 1.0 (0.8-1.5) | 0.04 |
| Alanine aminotransferase (U/L) | 96 | 21 (16-26) | 69 | 18 (14-22) | 0.03 |
| Erythrocyte sedimentation rate (mm/hour) | 95 | 11 (7-25) | 66 | 14 (7-21) | 0.64 |
| C-reactive protein (mg/L) | 97 | 5 (2-12) | 66 | 5 (2-9) | 0.15 |
| Immunoglobulin G level (g/L) | 93 | 6.9 (5.6-8.3) | 67 | 6.1 (4.7-7.8) | 0.04 |
| Immunoglobulin M level (g/L) | 95 | 0.4 (0.2-0.6) | 66 | 0.4 (0.2-0.7) | 0.77 |
| Immunoglobulin A level (g/L) | 95 | 1.6 (1.2-2.4) | 67 | 1.2 (0.9-1.8) | 0.003 |
| PR3 or MPO positive | 98 | 44 (45%) | 70 | 41 (59%) | 0.08 |
| PR3 positive | 98 | 29 (30%) | 70 | 27 (39%) | 0.22 |
| PR3 binding level (AI units) | 77 | 0.3 (0.1-1.4) | 53 | 0.3 (0.1-3.3) | 0.63 |
| MPO positive | 98 | 15 (15%) | 70 | 14 (20%) | 0.43 |
| MPO binding level (AI units) | 77 | 0.1 (0.1-0.1) | 53 | 0.1 (0.1-0.4) | 0.17 |
| CD19+ B-cells present (≥ 0.01 cells per 10^9^/L or ≥ 1%) | 90 | 7 (8%) | 59 | 6 (10%) | 0.61 |
| CD19+ B-cell count (10^9^/L) | 76 | 0 (0-0) | 42 | 0 (0-0) | 0.15 |
| CD19+ B-cells ≥ 0.01 cells per 10^9^/L | 76 | 4 (5%) | 42 | 5 (12%) | 0.19 |
| CD19+ B-cell percentage | 85 | 0 (0-0) | 56 | 0 (0-0) | 0.89 |
| CD19+ B-cell percent ≥ 1% | 85 | 6 (7%) | 56 | 3 (5%) | 0.69 |
| **Outcome measures** |  |  |  |  |  |
| BVAS/WG Score | 99 | 0 (0-0) | 70 | 0 (0-0) | 0.09 |
| Physician global assessment (0-10) | 99 | 0 (0-0) | 70 | 0 (0-0) | 0.41 |
| Patient global assessment (0-10) | 81 | 2 (0-3) | 60 | 2 (0-4) | 0.90 |
| EQ-5D Visual Analogue Scale (0-100)* | 97 | 75 (67-85) | 69 | 75 (60-87) | 0.78 |
| PROMIS Fatigue 4a T-score | 83 | 48.6 (46.0-57.0) | 59 | 51.0 (46.0-58.8) | 0.42 |
| PROMIS Pain Interference 4a T-score | 84 | 41.6 (41.6-57.1) | 61 | 49.5 (41.6-57.1) | 0.72 |
| PROMIS Physical Function 4a T-score* | 84 | 26.6 (22.5-34.4) | 61 | 28.9 (22.5-34.4) | 0.80 |
| Values presented as n (%) or median (IQR) unless otherwise specified. *Higher score indicates better health status. AI, antibody index; BVAS, Birmingham Vasculitis Activity Score/WG; CRP, C-reactive protein; EQ-5D, EuroQol-5 Dimension; MPO, myeloperoxidase; PR3, proteinase-3; PROMIS, Patient-Reported Outcomes Measurement Information System | | | | | |

| **Supplementary Table S2. Logistic generalized estimating equations models for odds of relapse by next visit for follow-up variables adjusted for the base model for each phase** | | | | |
| --- | --- | --- | --- | --- |
| **Models (base model + time-varying covariate)** | **Maintenance^†^** | | **Off-treatment ^⁰^** | |
|  | **OR (95% CI)** | **p-value** | **OR (95% CI)** | **p-value** |
| **Laboratory values** |  |  |  |  |
| Hemoglobin (g/L)^a^ | 0.94 [0.68, 1.31] | 0.73 | 1.21 [0.89, 1.63] | 0.22 |
| Platelet count (10^9^/L)^b^ | 0.93 [0.70, 1.22] | 0.59 | 1.22 [0.83, 1.79] | 0.30 |
| White blood cell count (10^9^/L)^a^ | 1.02 [0.77, 1.35] | 0.90 | 0.97 [0.63, 1.49] | 0.89 |
| Creatinine (mg/dl) ^b^ | 0.76 [0.55, 1.05] | 0.10 | 0.79 [0.57, 1.11] | 0.18 |
| Alanine aminotransferase (U/L)^b^ | 1.02 [0.73, 1.42] | 0.91 | 0.93 [0.69, 1.24] | 0.62 |
| Erythrocyte sedimentation rate (mm/hr)^b^ | 1.00 [0.76, 1.30] | 0.98 | 1.17 [0.87, 1.59] | 0.30 |
| C-reactive protein (mg/L)^b^ | 1.19 [0.85, 1.68] | 0.32 | 2.00 [1.36, 2.94] | <0.001 |
| Immunoglobulin G level (g/L)^a^ | 1.20 [0.94, 1.54] | 0.15 | 1.22 [0.75, 1.96] | 0.43 |
| Immunoglobulin M level (g/L)^b^ | 1.16 [0.86, 1.57] | 0.32 | 1.17 [0.83, 1.66] | 0.37 |
| Immunoglobulin A level (g/L)^b^ | 1.04 [0.75, 1.44] | 0.82 | 1.89 [1.31, 2.73] | 0.001 |
| PR3 positive | 0.86 [0.41, 1.80] | 0.68 | 1.82 [0.93, 3.55] | 0.08 |
| MPO positive | 0.59 [0.17, 1.98] | 0.39 | 2.51 [0.63, 10.06] | 0.19 |
| PR3 or MPO positive | 0.77 [0.40, 1.47] | 0.42 | 1.97 [1.08, 3.61] | 0.03 |
| MPO or PR3 binding level (AI) | 0.95 [0.85, 1.07] | 0.39 | 1.11 [1.01, 1.22] | 0.03 |
| CD19+ B-cells present (≥ 0.01 cells per 10^9^/L or ≥ 1%) | 1.65 [0.70, 3.93] | 0.25 | 2.47 [1.21, 5.06] | 0.02 |
| **Outcome measures** |  |  |  |  |
| Patient global assessment (0-10) | 1.12 [1.00, 1.24] | 0.04 | 0.99 [0.88, 1.10] | 0.81 |
| EQ-5D Visual Analogue Scale (0-100)^b^ | 0.95 [0.59, 1.53] | 0.83 | 1.28 [0.93, 1.77] | 0.13 |
| PROMIS Fatigue T-score^b^ | 1.17 [0.83, 1.64] | 0.37 | 0.96 [0.74, 1.26] | 0.78 |
| PROMIS Pain Interference T-score^b^ | 1.30 [0.97, 1.73] | 0.08 | 0.94 [0.69, 1.27] | 0.68 |
| PROMIS Physical Function T-score^b^ | 1.09 [0.82, 1.47] | 0.54 | 0.85 [0.61, 1.19] | 0.34 |
| **Other** |  |  |  |  |
| Time since prior rituximab, months | 1.03 [0.96, 1.10] | 0.39 | 0.98 [0.93, 1.03] | 0.37 |
| Body Mass Index (kg/m^2^)^b^ | 1.05 [0.79, 1.40] | 0.72 | 1.17 [0.88, 1.57] | 0.28 |
| **Change in laboratory values from prior visit** | | | | |
| ANCA change from prior visit (ref . negative to negative) |  |  |  |  |
| Negative to positive | 0.86 [0.18, 3.99] | 0.85 | 3.18 [1.31, 7.71] | 0.01 |
| Positive to positive | 0.72 [0.35, 1.48] | 0.37 | 1.62 [0.83, 3.16] | 0.16 |
| Positive to negative | 1.08 [0.40, 2.88] | 0.88 | 0.58 [0.07, 4.87] | 0.62 |
| CD19+ B-cell return (≥ 1% or ≥ 0.01 cells per 10^9^/L) compared to prior visit | 1.80 [0.56, 5.76] | 0.33 | 1.81 [0.84, 3.90] | 0.13 |

(continued on next page)

| **Supplementary Table S2 (continued).** |  | |  | |
| --- | --- | --- | --- | --- |
| **Models (base model + time-varying covariate)** | **Maintenance^†^** | | **Off-treatment ^⁰^** | |
|  | **OR (95% CI)** | **p-value** | **OR (95% CI)** | **p-value** |
| **Percent change in laboratory values** | | | | |
| Hemoglobin (g/L)^c^ | 0.92 [0.62, 1.35] | 0.66 | 1.04 [0.79 1.38] | 0.77 |
| Platelet count (10^9^/L)^c^ | 0.98 [0.84, 1.16] | 0.85 | 1.26 [1.04, 1.53] | 0.02 |
| White blood cell count (10^9^/L)^c^ | 1.01 [0.96, 1.05] | 0.77 | 1.04 [1.01, 1.08] | 0.02 |
| Creatinine (mg/dl)^c^ | 1.09 [0.83, 1.42] | 0.54 | 0.96 [0.81, 1.14] | 0.66 |
| Alanine aminotransferase (U/L)^c^ | 0.99 [0.97, 1.01] | 0.46 | 0.97 [0.92, 1.02] | 0.25 |
| Erythrocyte sedimentation rate (mm/hr)^c^ | 1.01 [0.99, 1.03] | 0.37 | 1.01 [0.99, 1.03] | 0.34 |
| C-reactive protein (mg/L)^c^ | 1.00 [1.00, 1.00] | 0.52 | 1.01 [1.00, 1.01] | 0.03 |
| Immunoglobulin G level (g/L)^c^ | 1.07 [0.96, 1.20] | 0.23 | 1.02 [0.97, 1.08] | 0.46 |
| Immunoglobulin M level (g/L)^c^ | 1.00 [0.98, 1.02] | 0.80 | 1.00 [0.97, 1.04] | 0.89 |
| Immunoglobulin A level (g/L)^c^ | 1.01 [0.98, 1.05] | 0.51 | 1.16 [1.08, 1.25] | <0.001 |
| **Percent change in outcome measures** | | | | |
| Patient global assessment (0-10)^c^ | 1.02 [0.98, 1.05] | 0.34 | 1.01 [0.98, 1.04] | 0.59 |
| EQ-5D Health Scale (0-100)^c^ | 1.00 [0.97, 1.02] | 0.67 | 1.03 [0.86, 1.23] | 0.75 |
| PROMIS Fatigue T-score^c^ | 1.10 [0.92, 1.32] | 0.28 | 1.00 [0.85, 1.189] | 0.96 |
| PROMIS Pain Interference T-score^c^ | 1.02 [0.85, 1.22] | 0.85 | 1.05 [0.86, 1.28] | 0.65 |
| PROMIS Physical Function T-score^c^ | 0.97 [0.77, 1.22] | 0.81 | 0.95 [0.78, 1.15] | 0.60 |
| ^a^values standardized to month 4; ^b^values log-transformed and standardized to month 4; ^c^percent change compared to month 4 (remission), odds ratio scaled to represent 10% change in value; †Maintenance phase base model includes treatment, ANCA type, disease duration, joint involvement; ⁰Observation phase base model includes treatment, ANCA type, study month, and treatment by month interaction; Abbreviations: ANCA, anti-neutrophil cytoplasmic antibody; MPO, myeloperoxidase; PR3, proteinase-3; PROMIS, Patient Reported Outcomes Information System | | | | |

**Supplementary References**

1. Cartin-Ceba R, Indrakanti D, Specks U, et al. The Pharmacogenomic Association of Fcγ Receptors and Cytochrome P450 Enzymes With Response to Rituximab or Cyclophosphamide Treatment in Antineutrophil Cytoplasmic Antibody–Associated Vasculitis. *Arthritis Rheumatol*. 2017;69(1):169-175. doi:https://doi.org/10.1002/art.39822

2. Kaul R, Johnson K, Scholz H, Marr G. Performance of the BioPlex 2200 Autoimmune Vasculitis kit. *Autoimmun Rev*. 2009;8(3):224-227. doi:10.1016/j.autrev.2008.07.033

3. Gopaluni S, Smith RM, Lewin M, et al. Rituximab versus azathioprine as therapy for maintenance of remission for anti-neutrophil cytoplasm antibody-associated vasculitis (RITAZAREM): study protocol for a randomized controlled trial. *Trials*. 2017;18(1). doi:10.1186/S13063-017-1857-Z
